# In vivo itaconate tracing reveals degradation pathway and turnover kinetics

**DOI:** 10.1038/s42255-025-01363-1

**Published:** 2025-09-10

**Authors:** Hanna F. Willenbockel, Alexander T. Williams, Alfredo Lucas, Mack B. Reynolds, Emeline Joulia, Maureen L. Ruchhoeft, Birte Dowerg, Pedro Cabrales, Christian M. Metallo, Thekla Cordes

**Affiliations:** 1 Department of Bioinformatics and Biochemistry, Braunschweig Integrated Centre of Systems Biology (BRICS), Technische Universität Braunschweig, Braunschweig, Germany; 2 Research Group Cellular Metabolism in Infection, Helmholtz Centre for Infection Research, Braunschweig, Germany; 3 Department of Bioengineering, University of California San Diego, La Jolla, CA USA; 4 Molecular and Cell Biology Laboratory, The Salk Institute for Biological Studies, La Jolla, CA USA

**Keywords:** Cell biology, Biochemistry, Physiology, Metabolism

## Abstract

Itaconate is an immunomodulatory metabolite that alters mitochondrial metabolism and immune cell function. This organic acid is endogenously synthesized by tricarboxylic acid (TCA) metabolism downstream of TLR signalling. Itaconate-based treatment strategies are under investigation to mitigate numerous inflammatory conditions. However, little is known about the turnover rate of itaconate in circulation, the kinetics of its degradation and the broader consequences on metabolism. By combining mass spectrometry and in vivo ^13^C itaconate tracing in male mice, we demonstrate that itaconate is rapidly eliminated from plasma, excreted via urine and fuels TCA cycle metabolism specifically in the liver and kidneys. Our results further reveal that itaconate is converted into acetyl-CoA, mesaconate and citramalate. Itaconate administration also influences branched-chain amino acid metabolism and succinate levels, indicating a functional impact on succinate dehydrogenase and methylmalonyl-CoA mutase activity in male rats and mice. Our findings uncover a previously unknown aspect of itaconate metabolism, highlighting its rapid catabolism in vivo that contrasts findings in cultured cells.

## Abstract (web summary)

In this study, Willenbockel et al. trace circulating itaconate in vivo to gain insight into its fate and systemic metabolism.

## Main

The small molecule itaconate is endogenously synthesized by immune cells, and the dynamic control of itaconate modulates metabolic pathways and immune responses^1–3^. Itaconate functions as a competitive inhibitor of succinate dehydrogenase (SDH), thereby influencing mitochondrial metabolism and immune cell function^4,5^. Despite extensive research on the biosynthetic pathway catalysed by aconitate decarboxylase (ACOD1), also known as immune-responsive gene 1 protein (IRG1), the itaconate degradation pathway remains poorly understood^6,7^. Itaconate is metabolized to itaconyl-coenzyme A (CoA)^8–10^, mesaconate^11–13^ and citramalyl-CoA^9,11^. Furthermore, degradation into pyruvate and acetyl-CoA was documented in liver mitochondria in the early 1960s^14,15^. However, in prior ^13^C itaconate tracer studies, we were unable to detect labelling on TCA cycle intermediates in cultured mammalian cell models, including those of the brain, immune system and hepatocytes, suggesting that itaconate is not metabolized to acetyl-CoA in cultured cells^11,16,17^. Itaconate has been applied therapeutically to mitigate the consequences of inflammatory stress in vivo^16,18,19^. However, the consequences of high circulating concentrations and the kinetics of its degradation in vivo remain unclear. To address this limitation, we performed ^13^C itaconate tracing in vivo and quantified itaconate dissimilation pathways and turnover rates.

### Dynamic itaconate metabolism and correlation with succinate levels in infused rat models

**Fig. 1:**
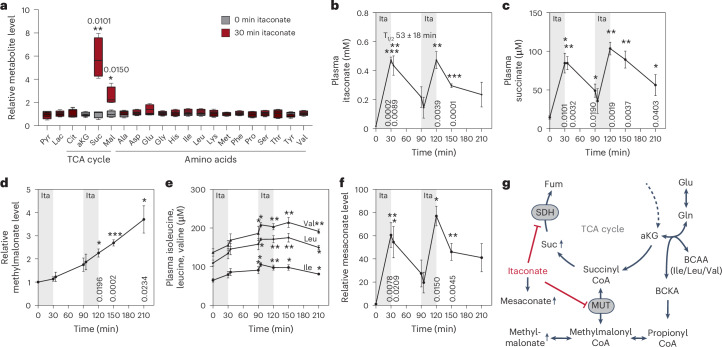
Dynamic itaconate metabolism and correlation with succinate levels in infused rat models. **a**, Plasma metabolite abundances at 30 min compared to 0 min after itaconate infusion. **b**, Plasma itaconate levels over time with two 30 min itaconate infusions (indicated in grey). **c**, Plasma succinate levels over time with two itaconate infusions. **d**, Relative level of methylmalonate in plasma over time with two itaconate infusions. **e**, Plasma level of leucine, isoleucine and valine over time with two itaconate infusions. **f**, Relative mesaconate level over time with two itaconate infusions. **g**, Schematic depicting the impact of itaconate on SDH and MUT activities. Experiments were performed with *n* = 4 rats; itaconate infusion (15 mg kg^−1^ min^−1^ for 30 min) was performed twice (indicated in grey). Data are presented as boxplots (25^th^ to 75^th^ percentile with median line) and whiskers (min. to max. values) (**a**) or mean ± s.e.m. (**b**–**f**) obtained from *n* = 4 rats. *P* values were calculated by multiple paired *t*-tests (**a**) or two-way ANOVA compared to 0 min with Fisher’s least significant difference (LSD) post hoc test (**b**–**f**); *P < 0.05; **P < 0.01; ***P < 0.001. Exact *P* values are indicated in each figure panel or in Extended Data Fig. 1f–h (**e**). Source data

**Extended Data Fig. 1:**
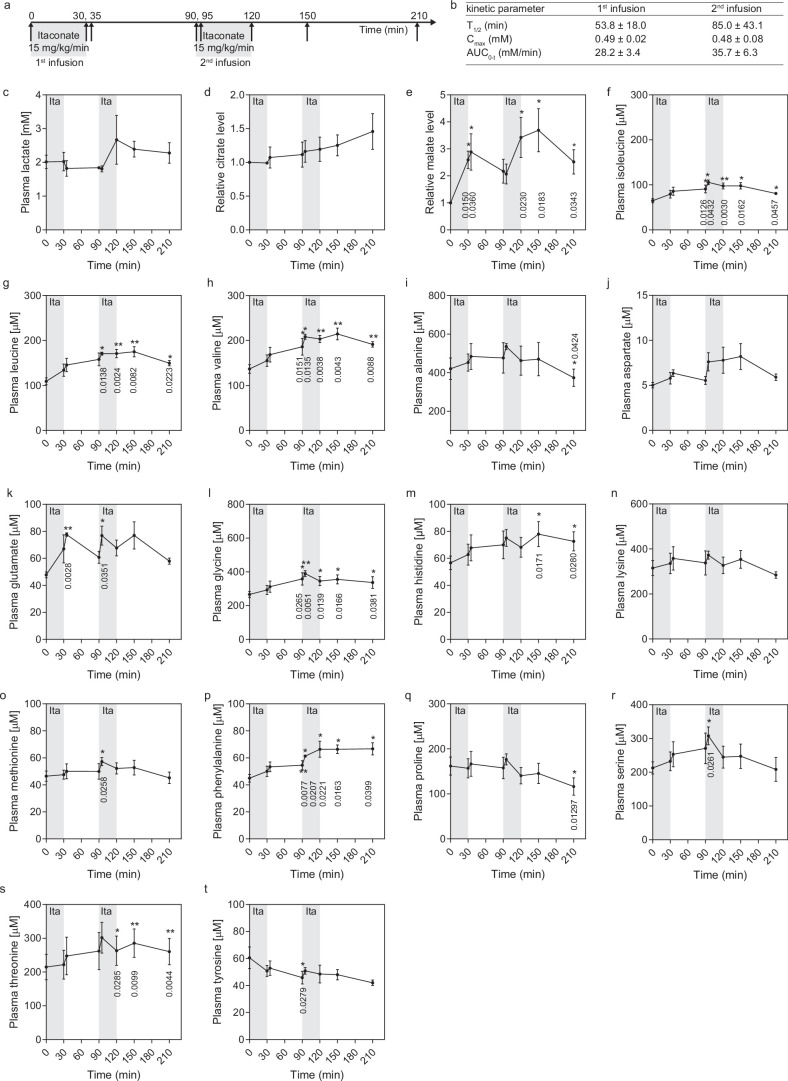
Plasma metabolite levels after itaconate infusion. **a**, Timeline of itaconate infusion and sample collection with two infusion cycles. **b**, Kinetic parameters for the two infusion cycles. **c**, Plasma lactate levels. **d**, Relative plasma citrate level. **e-t**, Plasma metabolite levels over time of **e**, malate, **f**, isoleucine, **g**, leucine, **h**, valine, **i**, alanine, **j**, aspartate, **k**, glutamate, **l**, glycine, **m**, histidine, **n**, lysine, **o**, methionine, **p**, phenylalanine, **q**, proline, **r**, serine, **s**, threonine, and **t**, tyrosine. T_1/2_ - elimination halftime; C_max_ - maximal plasma concentration; AUC - area under the curve. Experiments were performed with *n* = 4 rats and itaconate was infused twice with 15 mg kg^−1^ min^−1^ for each 30 min indicated in grey. Data are presented as means ± s.e.m. obtained from *n* = 4 rats. *P* values were calculated by two-way ANOVA compared to 0 min with Fisher’s least significant difference (LSD) post hoc test with *P < 0.05, **P < 0.01. Exact *p*-values are indicated in each figure panel. Source data

Itaconate accumulates at high levels in cells, but levels detected in circulation are in the µM range^20^. To increase systemic itaconate levels, in a previous study, we established an itaconate treatment strategy in an animal model of ischaemia–reperfusion and demonstrated that itaconate mitigates cellular injuries associated with reoxygenation^16^. We observed that itaconate was rapidly cleared from plasma and introduced a model in which itaconate transiently inhibits SDH activity to mitigate reperfusion-induced overactivation of SDH. Given that itaconate-induced SDH inhibition is reversible, itaconate levels might be fine-tuned to modulate cellular metabolism and function^16^. To gain more quantitative insights into the dynamics of itaconate metabolism, we infused male rat animal models with 15 mg kg^−1^ min^−1^ itaconate for 30 min and subsequently quantified plasma metabolite levels over time (Extended Data Fig. 1a). Infusion with itaconate for 30 min significantly elevated plasma concentrations of the TCA cycle intermediates succinate and malate, while other metabolites were less affected (Fig. 1a). These data indicate that TCA cycle metabolism might be the primary target of short-term itaconate treatments by modulating SDH activity. Next, we conducted a time-series analysis of plasma metabolite levels during two itaconate infusions, each lasting 30 min (Extended Data Fig. 1a). The plasma concentration of itaconate increased to approximately 0.45 mM, and most itaconate was cleared within 60 min. The second administration of itaconate yielded comparable outcomes, indicating that in vivo itaconate metabolism is highly dynamic (Fig. 1b). Next, we calculated the pharmacokinetic parameters for itaconate and observed an elimination half-time (*T*_½_) of 53 min following the initial infusion and 85 min following the second infusion (Fig. 1b and Extended Data Fig. 1b). Our kinetic parameters indicate that itaconate is rapidly cleared in vivo, suggesting a dynamic and reversible impact of itaconate on metabolism. Indeed, we observed that succinate concentrations correlated strongly with itaconate levels in the plasma, indicating reversible SDH activity inhibition (Fig. 1c). Itaconate also affected abundances of other TCA cycle intermediates, specifically malate, suggesting that itaconate-induced SDH inhibition may have additional effects on TCA cycle metabolism and related amino acids (Extended Data Fig. 1c–e).

Itaconate is metabolized to itaconyl-CoA, an inhibitor of both methylmalonyl-CoA mutase (MUT) and aminolevulinate synthase (ALAS), influencing mitochondrial coenzyme B_12_ and haem metabolism^8–10^. Impaired MUT activity leads to elevated levels of methylmalonate (MMA) and altered branched-chain amino acid (BCAA) metabolism^8,9,17^. In contrast to succinate levels, plasma MMA levels demonstrated a gradual accumulation over time, indicating itaconate-induced MUT inactivation (Fig. 1d). Furthermore, we observed that the levels of BCAAs leucine, isoleucine and valine were significantly increased over time in response to itaconate treatments (Fig. 1e and Extended Data Fig. 1f–h). These data suggest that the impact on BCAA metabolism may be less dynamic compared to succinate levels. Our observation further supports our previous in vitro studies indicating that itaconate-derived itaconyl-CoA irreversibly inactivates mitochondrial MUT activity, leading to increased MMA levels and altered BCAA metabolism^8,9,17^. In contrast to BCAAs, other plasma amino acids were less affected over time in response to itaconate (Extended Data Fig. 1i–t). Moreover, we observed a significant correlation between levels of plasma mesaconate, a metabolite synthesized from itaconate, and those of itaconate and succinate (Fig. 1b,c,f)^11,13^. Our study revealed that itaconate is rapidly cleared in vivo and consequently impacts mitochondrial and BCAA metabolism, indicating altered SDH and MUT activities (Fig. 1g).

### Itaconate is a substrate for mitochondrial metabolism in mouse liver and kidney

**Fig. 2:**
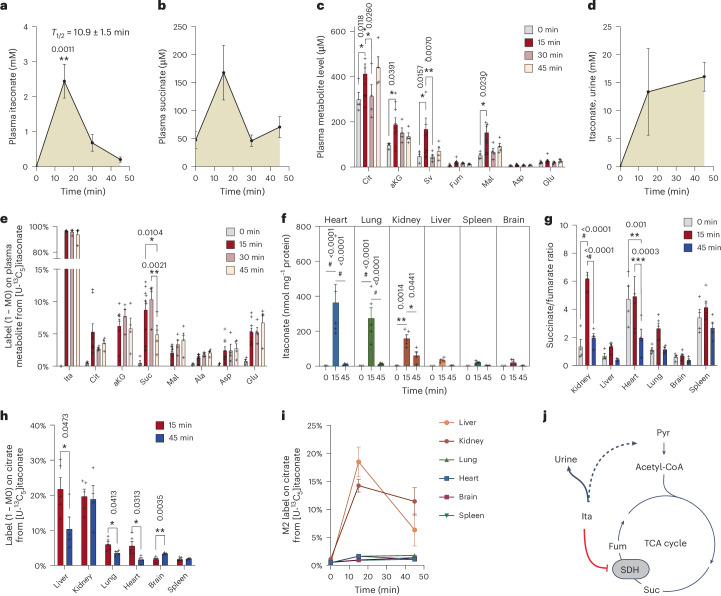
Itaconate is a substrate for mitochondrial metabolism in mouse liver and kidney. **a**, Plasma itaconate levels in response to itaconate treatment with elimination half-time (*T*_1/2_). **b**, Plasma succinate levels in response to itaconate treatment. **c**, Levels of TCA cycle intermediates after itaconate treatment for indicated times. **d**, Itaconate levels in urine. **e**, Labelling (1 − M0) on TCA cycle intermediates in plasma from [U-^13^C_5_]itaconate. **f**, Levels of itaconate in different tissues. **g**, Ratio of succinate (nmol mg^−1^ protein) over fumarate (nmol mg^−1^ protein) after itaconate treatment in different tissues. **h**, Label (1 − M0) on citrate from [U-^13^C_5_]itaconate in different tissues. **i**, M2 labelling on citrate from [U-^13^C_5_]itaconate in different tissues. **j**, Schematic depicting itaconate clearance pathway and SDH inhibition. Stashed arrows depict indirect metabolic pathways. Mice were injected with 400 mg kg^−1^ body weight [U-^13^C_5_]itaconate. Data are presented as means; error bars, s.e.m. Plasma samples at 0 min (*n* = 4), 15 min (*n* = 9), 30 min (*n* = 4) and 45 min (*n* = 4); tissue samples at 0 min (*n* = 4), 15 min (*n* = 5) and 45 min (*n* = 4); urine samples at 0 min (*n* = 3), 15 min (*n* = 4) and 45 min (*n* = 3). *P* values were calculated by one-way ANOVA compared to 0 min itaconate with Fisher’s LSD post hoc test (**a**,**b**,**d**) or two-way ANOVA (**c**,**e**–**h**); *P < 0.05; **P < 0.01, ***P < 0.001, ^#^*P* < 0.0001. Exact *P* values are indicated in each figure panel. Source data

**Extended Data Fig. 2:**
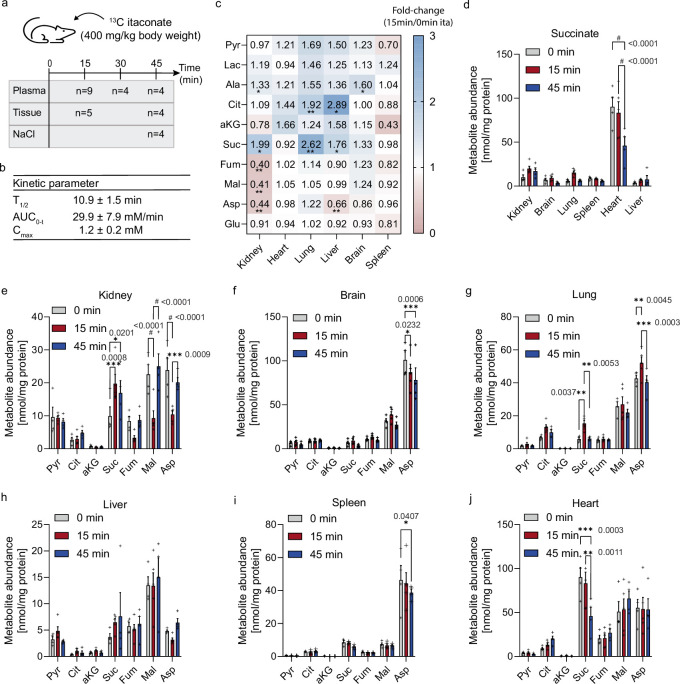
Itaconate influences abundances of TCA cycle intermediates in tissues. **a**, Schematic depicting an experimental overview of ^13^C itaconate tracing in mice with time points of tissue and plasma collections. **b**, Kinetic parameters after itaconate injection. **c**, Heat map depicting fold change of TCA cycle intermediate abundances 15 min after itaconate injection relative to control injection in diverse tissues. **d**, Succinate levels in different tissues. **e-j**, Levels of metabolites in **e**, kidney, **f**, brain, **g**, lung, **h**, liver, **i**, spleen, and **j**, heart. T_1/2_ - elimination halftime; C_max_ - maximal plasma concentration; AUC - area under the curve. Mice were injected with 400 mg kg^−1^ body weight [U-^13^C_5_]itaconate. Data are presented as heatmap (**c**) or means ± s.e.m. (**d**-**j**) with tissue samples at 0 min (*n*= 4), 15 min (*n* = 5), and 45 min (*n* = 4). *P* values were calculated by multiple unpaired *t*-test (**c**) or two-way ANOVA with Fisher’s least significant difference (LSD) post hoc test (**d**-**j**) with *P < 0.05, **P < 0.01, ***P < 0.001, ^#^*P* < 0.0001. Exact *P*-values are indicated in each figure panel. Source data

**Extended Data Fig. 3:**
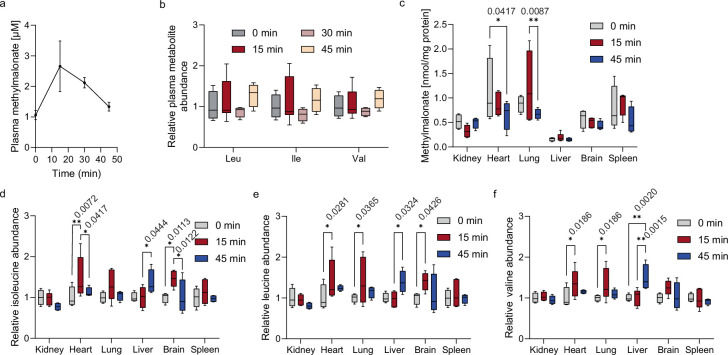
Itaconate modulates branched-chain amino acid metabolism in tissues. **a**, Plasma methylmalonate levels. **b**, Levels of valine, leucine, and isoleucine relative to 0 min itaconate. **c**, Levels of methylmalonate in different mouse tissues. Abundances of **d**, isoleucine, **e**, leucine, and **f**, valine relative to 0 min itaconate in different mouse tissues. Mice were injected with 400 mg kg^−1^ body weight [U-^13^C_5_]itaconate. Data are presented as means ± s.e.m. (**a**) or box (25^th^ to 75^th^ percentile with median line) and whiskers (min. to max. values) (**b**-**f**). Plasma samples at 0 min (*n* = 4), 15 min (*n* = 9), 30 min (*n* = 4), and 45 min (*n* = 4); tissue samples at 0 min (*n* = 4), 15 min (*n* = 5), and 45 min (*n* = 4). *P* values were calculated by one-way ANOVA compared to 0 min itaconate (**a**) or two-way ANOVA (**b**-**f**) with Fisher’s least significant difference (LSD) post hoc test with *P < 0.05, **P < 0.01. Exact *P*-values are indicated in each figure panel. Source data

Given that itaconate is rapidly cleared from plasma (Fig. 1b), we postulated that it might be taken up and further metabolized by tissues. To better understand the fate of itaconate, we applied a ^13^C itaconate tracer and quantified itaconate fluxes in vivo. Specifically, we administered a dose of 400 mg kg^−1^ body weight [U-^13^C_5_]itaconate to male mice and quantified metabolite abundances and labelling over time on plasma and tissue metabolome (Extended Data Fig. 2a). We observed that 15 min after itaconate administration, plasma itaconate levels increased to about 2.5 mM and were cleared within 45 min with *T*_½_ = 10.9 min (Fig. 2a and Extended Data Fig. 2b). We also observed a robust positive correlation between succinate and itaconate plasma levels in mice over time (Fig. 2b), indicating functional involvement of SDH activity. The abundances of other TCA cycle intermediates, such as malate, also correlated with itaconate levels, indicating a dynamic impact of itaconate on mitochondrial TCA cycle metabolism as previously observed in our rat model (Figs. 1 and 2c). Furthermore, we quantified a potential impact on MUT activity and observed that levels of MMA and BCAAs in the plasma were not affected after a short time upon itaconate treatments (Extended Data Fig. 3a,b). However, MMA and BCAAs increased in some tissues, suggesting that itaconate may affect Vitamin B_12_ metabolism and MUT activity in the liver and the heart, as previously observed in our rat model with longer itaconate treatments (Extended Data Fig. 3c–f and Fig. 1).

Previous studies demonstrated that when itaconate was fed to dogs, approximately 25% of the itaconate could be recovered in the urine, suggesting that parts of itaconate might be metabolized by tissues^21^. Therefore, we elucidated potential clearance pathways for ^13^C itaconate in vivo and observed approximately 15 mM itaconate in the urine (Fig. 2d). These results suggest that renal excretion is a major pathway for itaconate clearance. We also observed dynamic labelling on plasma TCA cycle intermediates and related amino acids 15 min after tracer injection, indicating that itaconate is metabolized further and used for mitochondrial TCA cycle metabolism (Fig. 2e). To identify the impact of circulating itaconate on tissue metabolism and identify potential tissues involved in itaconate dissimilation, we quantified levels and labelling on TCA cycle-related metabolites in diverse tissues. Itaconate was detected in all tissues after 15 min, with the highest concentrations observed in the kidney, heart and lung and lower levels in the liver, brain and spleen (Fig. 2f). Although a moderate increase in succinate was observed in tissues, succinate to fumarate ratios in the kidney and liver were significantly elevated, indicating impaired SDH activity (Fig. 2g and Extended Data Fig. 2c–j). In the heart, the high levels of succinate probably prevented itaconate from effectively inhibiting SDH (Extended Data Fig. 2j). Itaconate labelled around 20% of the liver and kidney citrate pool and up to 10% in other tissues (Fig. 2h). Notably, the liver and kidney appear to be highly involved in the itaconate dissimilation pathway, as M2 labelling on citrate reached approximately 20%, while citrate labelling in other tissues remained below 5% (Fig. 2i). Collectively, these data indicate that itaconate is cleared from plasma by renal clearance and metabolized by mitochondrial metabolism in the liver and kidney (Fig. 2j).

### The fate of ^13^C itaconate in liver tissue

**Fig. 3:**
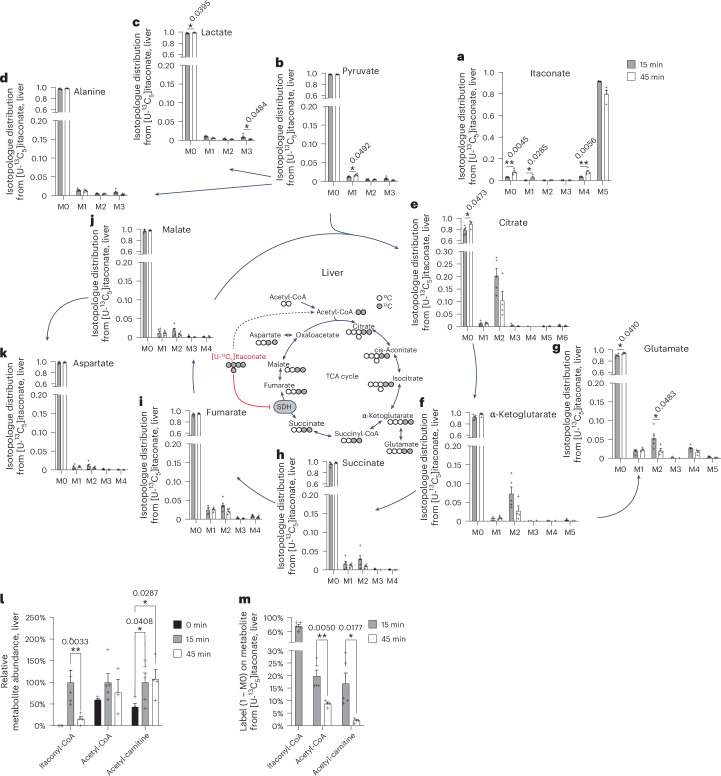
The fate of ^13^C itaconate in liver tissue. **a**–**k**, Isotopologue distribution from [U-^13^C_5_]itaconate on itaconate (**a**), pyruvate (**b**), lactate (**c**), alanine (**d**), citrate (**e**), α-ketoglutarate (**f**), glutamate (**g**), succinate (**h**), fumarate (**i**), malate (**j**) and aspartate (**k**) in liver tissue. **l**, Abundances of itaconyl-CoA, acetyl-CoA and acetyl-carnitine relative to 15 min baseline corrected to 100%. **m**, Labelling (1 − M0) on itaconyl-CoA, acetyl-CoA and acetyl-carnitine from [U-^13^C_5_]itaconate. In the schematic of [U-^13^C_5_]itaconate used for TCA cycle metabolism, open circles depict ^12^C and closed circles depict ^13^C. Mice were injected with 400 mg kg^−1^ body weight [U-^13^C_5_]itaconate. Data are presented as means; error bars, s.e.m. Tissue samples were collected at 0 min (*n* = 4), 15 min (*n* = 5) and 45 min (*n* = 4). *P* values were calculated by multiple unpaired *t*-tests (**a**–**k**, **m**) or two-way ANOVA with Fisher’s LSD post hoc test (**l**); *P < 0.05; **P < 0.01; ***P < 0.001. Exact *P* values are indicated in each figure panel. Source data

**Extended Data Fig. 4:**
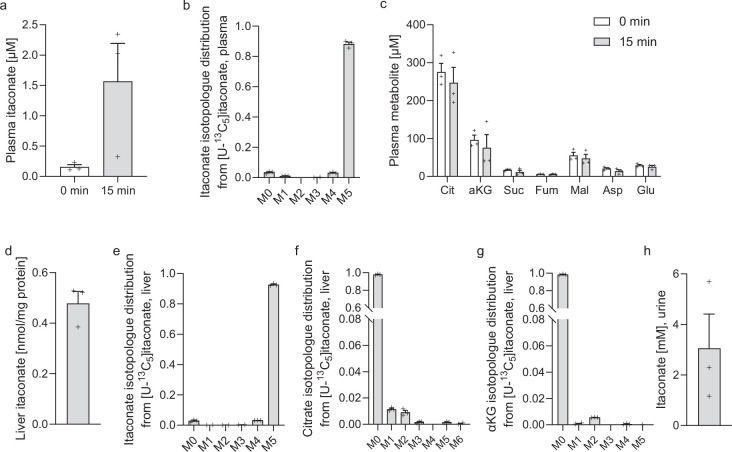
Low dose ^13 ^C itaconate treatment reveals itaconate dynamics in vivo. **a**, Plasma itaconate. **b**, Isotopologue distribution from [U-^13^C_5_]itaconate on plasma itaconate. **c**, Plasma metabolite levels. **d**, Itaconate levels in liver. Isotopologue distribution from [U-^13^C_5_]itaconate on liver **e**, itaconate **f**, citrate, and **g**, α-ketoglutarate. **h**, itaconate levels in the urine. Mice were injected with low - dose 40 mg kg^−1^ body weight [U-^13^C_5_]itaconate. Data are presented as means ± s.e.m. from *n* = 3 mice. Multiple paired *t*-test (**a**, **c**) with *P* > 0.05. Source data

Next, we quantified mass isotopomer distributions on TCA cycle intermediates from [U-^13^C_5_]itaconate to identify potential pathways involved in itaconate dissimilation. Given that citrate was the most heavily labelled metabolite in the liver tissue, we monitored the ^13^C-derived itaconate carbons in this tissue 15 min and 45 min after itaconate injection (Fig. 2i). We observed that the amount of fully labelled M5 itaconate was nearly 100%, indicating that the endogenous itaconate present in the tissue was negligible (Fig. 3a). Although the levels of pyruvate, lactate and alanine exhibited lower labelling, approximately 20% of M2 citrate was labelled (Fig. 3b–e). Furthermore, additional TCA cycle intermediates were robustly labelled with M2, including α-ketoglutarate, fumarate, succinate and malate, as well as related amino acids glutamate and aspartate (Fig. 3f–k). To test this activity under more physiological conditions, we performed low-dose ^13^C itaconate tracing. We observed a modest increase in labelled plasma itaconate, while abundances of TCA cycle intermediates remained unchanged 15 min after tracer administration (Extended Data Fig. 4a–c). In the liver, labelled itaconate was converted into M2-labelled citrate and α-ketoglutarate (Extended Data Fig. 4d–g). This tracing study further supports the usage of itaconate in TCA cycle metabolism under more physiological conditions with low itaconate plasma and tissue concentrations, while the majority of itaconate was excreted in the urine (Extended Data Fig. 4h).

**Extended Data Fig. 5:**
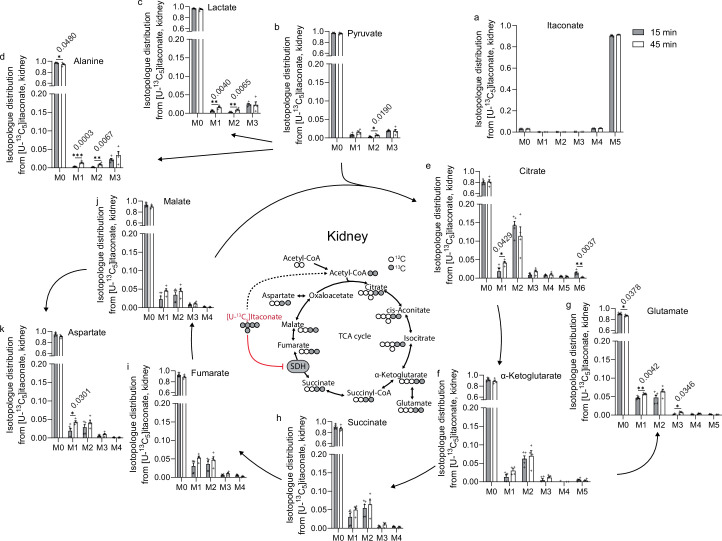
The fate of ^13 ^C itaconate in kidney tissue. **a-k** Isotopologue distribution from [U-^13^C_5_]itaconate on **a**, itaconate, **b**, pyruvate, **c**, lactate, **d**, alanine, **e**, citrate, **f**, α-ketoglutarate, **g**, glutamate, **h**, succinate, **i**, fumarate, **j**, malate, and **k**, aspartate in kidney tissue. Schematic of [U-^13^C_5_]itaconate use for TCA cycle metabolism with open circles depicting ^12^C and closed circles ^13^C. Mice were injected with 400 mg kg^−1^ body weight [U-^13^C_5_]itaconate. Data are presented as means ± s.e.m. with tissue samples at 0 min (*n* = 4), 15 min (*n* = 5), and 45 min (*n* = 4). *P* values were calculated by multiple unpaired *t*-test with *P < 0.05, **P < 0.01, ***P < 0.001. Exact *P*-values are indicated in each figure panel. Source data

These labelling patterns also indicate that itaconate is not converted to citrate through the reverse ACOD1 synthesis pathway, which would result in M5 on citrate. Conversely, itaconate is metabolized to a labelled C_2_ compound, which fuels the citrate pool, resulting in an M2 labelling on citrate (Fig. 3e). We quantified high itaconyl-CoA abundances in itaconate-treated liver tissue, while no itaconyl-CoA was detectable in control conditions, indicating that itaconyl-CoA is an intermediate of the itaconate dissimilation pathway (Fig. 3l). Given the small amount of labelling observed on M3 pyruvate, we proceeded to quantify acetyl-CoA levels, which demonstrated a modest increase in response to itaconate treatment (Fig. 3l). Furthermore, we detected elevated levels of acetyl-carnitine (Fig. 3l). ^13^C itaconate highly labelled itaconyl-CoA, approximately 20% of the acetyl-CoA pool, and comparable labelling was observed on acetyl-carnitine (Fig. 3m). These carbons may be used to form M2 labelling on citrate and other TCA cycle intermediates through the acetyl-CoA pathway. Thus, itaconate may serve as a carbon fuel for TCA cycle metabolism through acetyl-CoA, resulting in M2 labelling on citrate specifically in the kidney and liver (Fig. 2i). Indeed, the mass isotopologue distribution of TCA cycle intermediates in the kidney revealed the presence of M2 labelling on TCA cycle intermediates, while pyruvate, lactate and alanine exhibited M3 labelling derived from ^13^C itaconate (Extended Data Fig. 5). Therefore, itaconate may fuel TCA cycle metabolism through acetyl-CoA, particularly in the liver and kidney.

### Itaconate is metabolized to mesaconate and citramalate in vivo

**Fig. 4:**
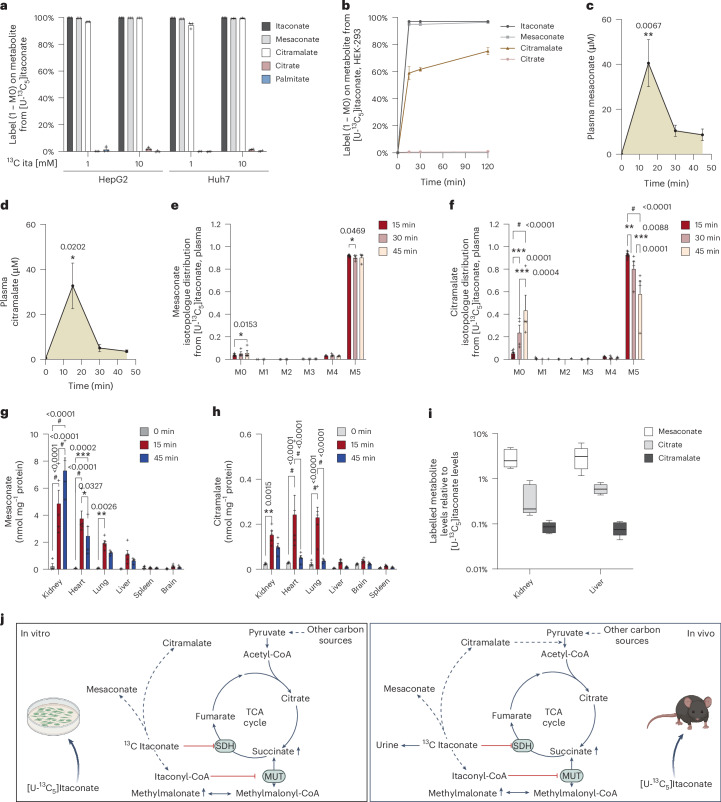
Itaconate is metabolized to mesaconate and citramalate in vivo. **a**, Labelling (1 − M0) on itaconate, mesaconate, citramalate, citrate and palmitate in HepG2 and Huh7 cells after 24 h culture with 1 mM and 10 mM [U-^13^C_5_]itaconate. **b**, Labelling (1 − M0) on itaconate, mesaconate, citramalate and citrate in HEK-293 cells cultured for 15, 30 and 120 min with 3 mM [U-^13^C_5_]itaconate. **c**, Plasma mesaconate level. **d**, Plasma citramalate level. **e**, Plasma mesaconate isotopologue distribution, **f**, Plasma citramalate isotopologue distribution. **g**, Labelled mesaconate abundance in tissues. **h**, Labelled citramalate abundance in tissues. **i**, Labelled metabolite (nmol mg^−1^ protein) relative to itaconate levels (nmol mg^−1^ protein) in liver and kidney tissues 15 min after ^13^C itaconate injection. **j**, Schematic depicting potential itaconate dissimilation in in vivo and in vitro models. Mice were injected with 400 mg kg^−1^ body weight [U-^13^C_5_]itaconate. Data are presented as mean ± s.e.m. (**a**–**h**) or box (25^th^ to 75^th^ percentile with median line) and whiskers (min. to max. values) (**i**). Plasma samples at 0 min (*n* = 4), 15 min (*n* = 9), 30 min (*n* = 4) and 45 min (*n* = 4); tissue samples at 0 min (*n* = 4), 15 min (*n* = 5). *P* values were calculated by one-way ANOVA compared to 0 min itaconate (**c**,**d**) or two-way ANOVA (**e**–**h**) with Fisher’s LSD post hoc test; *P < 0.05; **P < 0.01; ***P < 0.001; ^#^*P* < 0.0001; exact *P* values are indicated in each figure panel. Schematic in Fig. 4j created in BioRender.com. Source data

**Extended Data Fig. 6:**
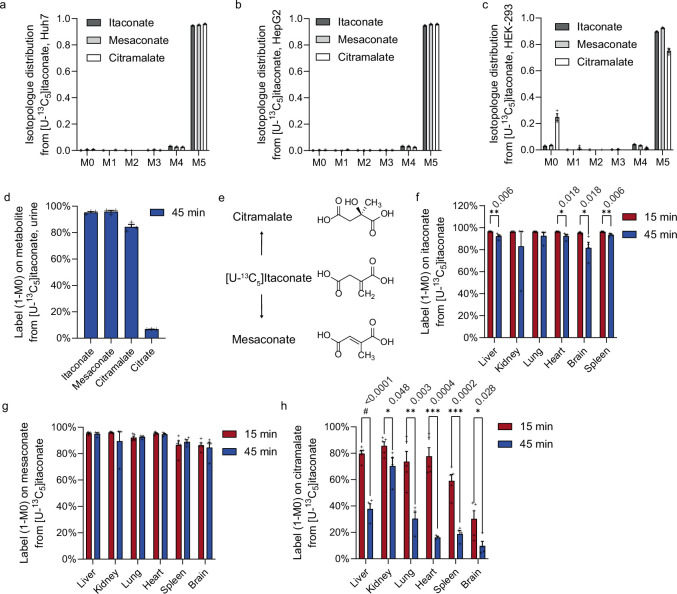
Itaconate labels citramalate and mesaconate in tissues and culture models. **a**, Isotopologue distribution from [U-^13^C_5_]itaconate on itaconate, mesaconate, and citramalate in Huh7 cells cultured with 10 mM ^13^C itaconate for 24 h, **b**, Isotopologue distributions on metabolites from HepG2 cells cultured with 10 mM ^13^C itaconate for 24 h, and **c**, HEK-293 cells cultured with 3 mM ^13^C itaconate for 120 min. **d**, Label on metabolites in the urine. **e**, Chemical structures of itaconate, mesaconate, and citramalate. **f**, Labeling (1 – M0) on itaconate **g**, mesaconate, and **h**, citramalate from [U-^13^C_5_]itaconate in different tissues. Mice were injected with 400 mg/kg body weight [U-^13^C_5_]itaconate. Data are presented as means ± s.e.m. Plasma samples at 0 min (*n* = 4), 15 min (*n* = 9), 30 min (*n* = 4), and 45 min (*n* = 4); tissue samples at 0 min (*n* = 4), and 15 min (*n* = 5). *P* values were calculated by multiple unpaired *t*-test (**f**-**h**) with *P < 0.05, **P < 0.01, ***P < 0.001, ^#^*P* < 0.0001. Exact *P*-values are indicated in each figure panel. Source data

In our previous studies, we traced various cell types, including immune cells, neurons, astrocytes and hepatocarcinoma cells, with ^13^C itaconate but did not detect labelling on citrate^11,16,17^. Given that these studies were performed with 2 mM or lower concentrations of ^13^C itaconate, we cultured the hepatocarcinoma cell lines HepG2 and Huh7 in the presence of 1 mM and 10 mM [U-^13^C_5_]itaconate for 24 h. Similar to our previous studies, we did not observe labelled citrate or palmitate, even after treatment with 10 mM ^13^C itaconate. These data suggest that itaconate is not metabolized to acetyl-CoA in vitro, which serves as a fuel for the TCA cycle metabolism and de novo lipogenesis (Fig. 4a). Therefore, the itaconate dissimilation pathway may exhibit differential behaviour in cells compared to in vivo conditions. Subsequently, we quantified the labelling of mesaconate and citramalate, two metabolites derived from itaconate^11,14,15,22^. We observed fully M5-labelled mesaconate and citramalate in both cell lines, indicating that all five carbons for the carbon backbone were derived from itaconate (Fig. 4a and Extended Data Fig. 6a,b). As our studies with male mice were conducted with shorter time points, we also quantified time-dependent metabolic fluxes and traced cells for 15, 30 and 60 min. Labelling of citrate was negligible, while mesaconate, citramalate and itaconate were fully labelled even at the 15 min time point (Fig. 4b and Extended Data Fig. 6c). We also observed that parts of the labelled mesaconate, citramalate and citrate were present in the urine, indicating renal clearance (Extended Data Fig. 6d). Therefore, itaconate provides the five-carbon backbone for the formation of mesaconate and citramalate, while itaconate-derived carbons also fuelled TCA cycle metabolism in our in vivo models (Extended Data Fig. 6e).

Given that mesaconate and citramalate are derived from itaconate, we quantified the kinetics of these C_5_ dicarboxylate compounds in vivo following itaconate treatment. Our study revealed a robust correlation between plasma levels of mesaconate and citramalate and those of itaconate (Fig. 4c,d). Mesaconate labelling indicates that it is directly derived from itaconate, and negligible endogenous mesaconate was present in these otherwise healthy animals (Fig. 4e and Extended Data Fig. 6f,g). By contrast, citramalate was also M5-labelled but the labelling decreased over time, indicating dynamic itaconate metabolism and the presence of endogenous, unlabelled citramalate (Fig. 4f and Extended Data Fig. 6h). Furthermore, mesaconate and citramalate were synthesized in a variety of tissues, with kidney showing the highest abundances (Fig. 4g,h). To gain further insight into the extent of itaconate metabolism to mesaconate, citramalate and citrate, we normalized the labelled abundances of these metabolites to labelled itaconate abundance at 15 min post injection that was present in kidney and liver tissues. We observed that approximately 5% of itaconate is converted to mesaconate, 1% to citrate and less than 1% to citramalate (Fig. 4i). Thus, circulating itaconate is excreted by renal clearance, and a small fraction undergoes further metabolism with involvement of kidney and liver. Furthermore, C_5_ dicarboxylate compounds mesaconate and citramalate are derived from itaconate in both in vivo and in vitro conditions, whereas conversion into citrate occurred in in vivo conditions only (Fig. 4j).

## Discussion

Our ^13^C itaconate tracing study elucidated the turnover kinetics and dissimilation pathways to provide insights into dynamic itaconate metabolism, pharmacokinetics and effects on TCA cycle and BCAA metabolism through the inhibition of SDH and MUT activity. We identified renal excretion as the major pathway for itaconate clearance, with a small fraction of itaconate being metabolized to mesaconate, citramalate and acetyl-CoA, fuelling TCA cycle metabolism. This metabolic fate of itaconate suggests a previously unidentified metabolic function for itaconate, linking itaconate to substrate use and metabolic regulation.

Our in vivo study demonstrates that itaconate levels correlate strongly with plasma succinate levels, suggesting a regulatory role for itaconate in systemic succinate levels and subsequent immune responses^23^. Given that SDH is complex II of the mitochondrial respiratory chain, this dynamic regulation of SDH may be beneficial in situations of limited oxygen availability, such as diseases associated with reoxygenation injury. Our in vivo data support the model that itaconate functions as a competitive and reversible SDH inhibitor, which gradually awakens mitochondrial flux and thereby mitigates reperfusion injury^5,16,17^. Although single doses of itaconate rapidly affected SDH function, effects on BCAA metabolism and methylmalonate were less reversible, potentially owing to MUT inactivation by itaconyl-CoA^8,17^. These findings further support our previous observation in cultured cell models that itaconate may affect transaminase reactions^17^. The long-term effects on BCAA and vitamin B_12_ metabolism may have implications in cell types with highly activated BCAA metabolism, such as adipocytes^24,25^.

Our tracing study reveals significant effects of itaconate on the TCA cycle and CoA metabolism in the liver. Itaconate labelled around 20% of the citrate pool at high doses and had detectable levels of citrate at more physiological doses. These data suggest that hepatic catabolism is an active route of disposal, particularly when itaconate is produced locally in inflamed tissue. However, itaconate in the bloodstream is predominantly excreted in urine. These findings are consistent with previous research demonstrating that itaconate exerts beneficial effects in metabolic disorders, including diet-induced obesity^18,26^ and non-alcoholic fatty liver disease^27^. Itaconate is transported in the liver by the sodium dicarboxylate cotransporter^26,28^. Therefore, circulating itaconate may impact hepatic metabolism, and our itaconate clearance data may provide insight into potential treatment strategies. Owing to its water-soluble nature, the addition of itaconate to drinking water^29^ or fluid therapies^16^ may facilitate long-term and continuous administration of itaconate. Further research is needed to elucidate the long-term effects of itaconate supplementation and optimize dosing regimens.

Itaconate has antimicrobial properties, and certain bacterial species have developed strategies to use itaconate as a carbon source for detoxification^30^. Therefore, the itaconate dissimilation pathway may be conserved across species, and further research is needed to elucidate potential discrepancies of the itaconate dissimilation pathway in different cell types and model systems, including ^13^C tracing in rat models. We investigated the metabolic consequences following itaconate treatment. However, a limitation of our study is that the physiological relevance of circulating itaconate remains incompletely understood. Further research is required to elucidate the role and significance of exogenously administered itaconate compared to endogenously synthesized itaconate. Another limitation of our current study is the use of male models, which precludes assessment of potential sex-specific differences in itaconate metabolism; future studies should address this gap by including female subjects. Additional investigation is also needed to identify potential metabolites and enzymes involved in this pathway. For example, enzymes involved in the synthesis of itaconyl-CoA, such as succinyl-CoA:glutarate-CoA transferase (SUGCT)^10^ and succinyl-CoA synthetase (SCS)^14,15^, may influence mesaconate synthesis, substrate phosphorylation and CoA homoeostasis^11,12,17^. Itaconyl-CoA might be hydrated to citramalyl-CoA and cleaved into acetyl-CoA and pyruvate by citrate lyase beta-like (CLYBL) protein activity in a tissue-dependent manner^9,14,15,31^. Thus, our in vivo tracing data highlight the involvement of the liver and kidneys, suggesting tissue-specific regulatory and transport mechanisms in the itaconate dissimilation pathway that may influence CoA homoeostasis and mitochondrial metabolism.

Although numerous studies have focused on the immunomodulatory effects of itaconate, the present study provides a distinct perspective on its role in regulating metabolism^3,32–34^. Itaconate also possesses beneficial properties in the context of cancer, obesity and other diseases^18,28,35–38^. Thus, some effects of itaconate may be attributed to itaconate degradation products and subsequent metabolic reprogramming that may be beneficial in diverse disease settings.

## Methods

### Animal studies

Animal handling and care followed the National Institutes of Health Guide for Care and Use of Laboratory Animals (Protocols S00149, S11306 and 21-00034). The housing facility was maintained on a 12 h light–dark cycle, with an ambient temperature (21 °C) and humidity ranging from 40% to 60%. The experimental protocol was approved by the Salk Institute for Biological Studies and the University of California San Diego Institutional Animal Care and Use Committee.

### Itaconate infusion experiments in male rat models

Itaconate infusion studies were performed in male Sprague–Dawley rats (Harlan Laboratories) weighing 200–250 g. Animals were anaesthetized with isoflurane in compressed room air (Drägerwerk) and placed on a heating pad to maintain core body temperature at 37 °C for the duration of the experiment. A femoral catheter was implanted, and itaconate was infused at 15 mg kg^−1^ min^−1^ for 30 min. The infusion was stopped for 60 min, and itaconate was then infused again for 30 min for a second round. Plasma samples were taken at time points as indicated in the text from *n* = 4 animals. Data for rat infusion experiments are depicted in Fig. 1 and Extended Data Fig. 1.

### ^13^C itaconate study in male mouse models

Male C57BL/6J mice (8 weeks old) were obtained from Jackson Laboratories. Mice were administered 400 mg kg^−1^ body weight itaconate by retroorbital injection^39^. Itaconate was prepared in NaCl and adjusted to pH 7.3. Mice were fasted for 6 h before itaconate injection. Plasma and tissues were collected at the indicated time points (Extended Data Fig. 2a) and further used for metabolite analysis. Animals were anaesthetized with isoflurane and decapitated, and the tissues were rapidly collected, frozen to temperatures of liquid nitrogen and stored at −80 °C until analysis. Tissues were collected from *n* = 5 animals for 15 min ^13^C itaconate treatment and *n* = 4 for 45 min ^13^C itaconate treatment. Plasma was collected at 15 min from all nine animals, while plasma at 30 min and 45 min was collected from four animals. Data were compared to the control condition (0 min itaconate), in which NaCl was given for 45 min from *n* = 4 animals. For the low-dose itaconate treatment depicted in Extended Data Fig. 4, 40 mg kg^−1^ body weight itaconate was administered by retroorbital injection to *n* = 3 animals. Liver tissues, plasma and urine were collected at 15 min after ^13^C itaconate administration, and plasma samples were compared to those obtained at 0 min ^13^C itaconate (before tracer administration).

### Cell culture

The cell lines HEK-293 (ATTC; CRL-1573), HepG2 (ATCC; HB-8065) and Huh7 (provided by M. Hermann, Massachusetts Institute of Technology) were used in the experiments. Cells were tested negative for mycoplasma contamination by the MycoAlert Mycoplasma Detection Kit (Lonza). Cells were cultured in DMEM (Gibco, cat. no. 11965-092) containing 25 mM glucose, 4 mM glutamine, 100 U ml^−1^ penicillin and 100 µg ml^−1^ streptomycin in a humidified cell culture incubator at 37 °C and 5 % CO_2_. Medium was supplemented with 10% FBS (Gibco, cat. no. 16000-044), and cells were detached with 0.05% trypsin-EDTA.

### Gas chromatography–mass spectrometry, sample preparation and analysis

Metabolites were extracted, analysed and quantified as previously described in detail^40^. In brief, plasma metabolite levels were extracted using 10 µl plasma and 90 µl methanol:water (8:1). Urine metabolites were extracted using 5 µl plasma and 45 µl methanol:water (8:1). Tissues were pulverized using a Cellcrusher cryogenic tissue pulverizer (Cellcrusher). The powder was stored at −80 °C until further use. A total of 10−20 mg of pulverized tissue was homogenized with a ball mill (Retsch Mixer Mill MM 400) at 30 Hz for 3 min, and metabolites were extracted with 0.5 ml of −20 °C methanol, 0.2 ml of cold water (4 °C) and 0.5 ml of −20 °C chloroform. A 50 µl aliquot was taken before chloroform addition to determine tissue protein content using the BCA protein assay (Lambda Biotech, G1002) for normalization. For absolute quantification of amino acids (Fig. 1 and Extended Data Fig. 1), 10 µl of 100 µM labelled (^13^C,^15^N) amino acid standard mix (MSK-A2-1.2, Cambridge Isotope Laboratories) was spiked to each sample (1 nmole per sample). Other metabolites, including TCA cycle intermediates, were quantified based on external standard curves. The plasma and tissue extracts were vortexed for 10 min at 4 °C and centrifuged at 16,000*g* for 5 min at 4 °C. The upper aqueous phase was evaporated under vacuum at 4 °C. Derivatization for polar metabolites was performed using a Gerstel MPS with 15 μl of 2 % (w/v) methoxyamine hydrochloride (Thermo Scientific) in pyridine (incubated for 60 min at 45 °C) and 15 μl *N*-tert-butyldimethylsilyl-*N*-methyltrifluoroacetamide with 1% tert-butyldimethylchlorosilane (Regis Technologies) (incubated for an additional 30 min at 45 °C). Derivatives were analysed by gas chromatography–mass spectrometry using a DB-35MSUI column (30 m × 0.25 internal diameter × 0.25 μm) installed in an Agilent 7890B gas chromatograph interfaced with an Agilent 5977A mass spectrometer operating under electron impact ionization at 70 eV. The mass spectrometer source was held at 230 °C, and the quadrupole at 150 °C; helium was used as the carrier gas. The gas chromatograph oven was held at 100 °C for 2 min, increased to 300 °C at 10 °C min^−1^ and held at 325 °C for 3 min.

The lower organic phase from Huh7 and HepG2 culture cells was derivatized to form fatty acid methyl esters (FAMEs) using 500 μl of 2% H_2_SO_4_ in methanol and incubation at 50 °C for 2 h. FAMEs were extracted by the addition of 100 μl saturated salt solution and 500 μl hexane. FAMEs were analysed using a Select FAME column (100 m × 0.25 mm internal diameter) installed in an Agilent 7890A gas chromatograph interfaced with an Agilent 5975C mass spectrometer. Helium was used as a carrier gas, and the gas chromatograph oven was held at 80 °C, increased by 20 °C min^−1^ to 170 °C, increased by 1 °C min^−1^ to 204 °C, then by 20 °C min^−1^ to 250 °C and held for 10 min.

### Measurements of CoA and carnitine species

Itaconyl-CoA, acetyl-CoA and acetyl-carnitine were measured using reverse-phase liquid chromatography, as described in our previous publication^17^. In brief, 10 mg pulverized tissue was homogenized with a ball mill (Retsch Mixer Mill MM 400) at 30 Hz for 5 min. Metabolites were extracted with 1 ml of −20 °C 80% methanol/water, and the extracts were centrifuged at 16,000*g* for 5 min at 4 °C. Then, 200 µl of the extracts were dried under airflow, resuspended in 100 μl of buffer A, and 5 µl of the sample was measured on a liquid chromatograph coupled to a Q Exactive system (Q Exactive Hybrid Quadrupole-Orbitrap mass spectrometer with a Vanquish Flex Binary UHPLC system; Thermo Scientific). A C18 column (C18 1.7 µm, 100 Å, 100 × 2.1 mm; Phenomenex, cat. no. 00D-4475-AN) was used with mobile phase buffer A (5 mM ammonium acetate in water, pH 6.8) and buffer B (100% methanol). The Q Exactive mass spectrometer was operated in positive mode. Metabolites were verified with external standards or specific MS2 fragments. Mass accuracy obtained for all metabolites was below 5 ppm. Data were acquired with Thermo Xcalibur software and analysed using EL-Maven software with correction for natural abundance^41^. Abundance was normalized to mg of tissue. Itaconyl-CoA was detected in itaconate-treated samples only and was M5-labelled from [U-^13^C_5_]itaconate.

### Isotopic tracing and analysis

Labelled [U-^13^C_5_]itaconate was provided by the Metabolite Standards Synthesis Core, arranged through the National Institutes of Health Common Fund’s Metabolomics Initiative^42^. Cells were cultured in growth medium containing 10% FBS and ^13^C itaconate. All media was adjusted to pH 7.3. Huh7 and HepG2 cells were cultured for 24 h in the presence of 1 mM and 10 mM ^13^C itaconate. HEK-293 cells were cultured for 15, 30 and 120 min with 3 mM ^13^C itaconate. Metabolites were extracted, and labelling on metabolites from ^13^C itaconate was quantified using gas chromatography–mass spectrometry. Mass isotopomer distributions and total metabolite abundances were computed by integrating mass fragments using a MATLAB-based algorithm with corrections for natural isotope abundances as described previously^40,43^. Labelling is depicted as 1 − M0 or isotopologue distribution as indicated in each figure.

### Plasma itaconate pharmacokinetics

Itaconate kinetic data analysis was performed using the add-in programme PKSolver 2.0 for Microsoft Excel^44^. Rat data depicted in Fig. 1 were generated through a non-compartmental analysis of plasma data after intravenous constant infusion for 30 min with an infusion dose of 15 mg kg^−1^ min^−1^ itaconate. Mouse data depicted in Fig. 2 were calculated through non-compartmental analysis of plasma data after intravenous bolus input with an infusion dose of 400 mg kg^−1^ body weight itaconate.

### Statistics

Data visualization and statistical analyses were performed using GraphPad Prism (v.10.3.1) and Adobe Illustrator CS6 (v.24.1.2). ChemDraw (v.23.1.1) was used for chemical structures. Figure 4j was created with BioRender.com. The type and number of replicates, number of animals (*n*) and the statistical tests used are described in each figure legend. Data are presented as means ± s.e.m. or box (25^th^ to 75^th^ percentile with median line) and whiskers (min. to max. values). *P* values were calculated using a two-sided *t*-test to compare two groups, one-way ANOVA or two-way ANOVA with Fisher’s least significant difference post hoc test. Samples were tested for normality using a Shapiro–Wilk test (*P* > 0.05). For all tests, *P* < 0.05 was considered significant, with *P* < 0.05, **P < 0.01, ***P < 0.001 and ^#^*P* < 0.0001 as indicated in each figure legend. No statistical methods were used to predetermine sample size. Sample sizes were determined based on our and other investigators’ experiences with the respective cell lines and animals used. The sample sizes were found to be adequate based on the magnitude and consistency of measurable differences between groups. No data were excluded from analysis. For in vitro experiments and mass spectrometry, all samples were randomly allocated into conditions. For in vivo experiments, all animals were randomly assigned to treatment groups. The investigators were not blinded to experimental conditions. The data reported for the experiments were based on quantitative cellular and metabolic measurements that are not subject to biases.

### Reporting summary

Further information on research design is available in the Nature Portfolio Reporting Summary linked to this article.

## Supplementary information

Reporting Summary

## Source data

Source Data Fig. 1 Source data and statistical source data.

Source Data Fig. 2 Source data and statistical source data.

Source Data Fig. 3 Source data and statistical source data.

Source Data Fig. 4 Source data and statistical source data.

Source Data Extended Data Fig. 1 Source data and statistical source data.

Source Data Extended Data Fig. 2 Source data and statistical source data.

Source Data Extended Data Fig. 3 Source data and statistical source data.

Source Data Extended Data Fig. 4 Source data and statistical source data.

Source Data Extended Data Fig. 5 Source data and statistical source data.

Source Data Extended Data Fig. 6 Source data and statistical source data.

## Data Availability

All data associated with this study are provided in the paper or Extended Data. Source data are deposited in the repository platform of Technische Universität Braunschweig (10.24355/dbbs.084-202505061250-0)^45^. Source data are provided with this paper.
